# Remarks, Gleanings, and Extracts

**Published:** 1874-03

**Authors:** R. C. Word

**Affiliations:** Georgia


### REMARKS, GLEANINGS AND EXTRACTS.

BY R. C. WORD, M.D., OF GEORGIA.

*Correction.*—In the article on *Hemorrhage in Abortion* in last issue of this Journal, the types make us say that the operation thus described was performed as far back as 1836, it should read 1856.

*The Brain During Sleep.*—Claud Bernard has recently contributed to the *Revue des Deux Mondes*, a valuable paper "On the Functions of the Brain," which has been translated from the November number of the *Popular Science Monthly*. Sleep, as he remarks, "is rightly considered the state of rest of the cerebral organ;" and he proceeds to give the following interesting account of the experiments by which it has been proved that sleep is not the result of compression produced by the accumulation of blood in the brain, as was commonly believed until within a few years:—

"It has been shown by direct experiment that, during sleep, the brain, instead of being congested, is on the contrary pale and bloodless; while in a state of wakefulness the circulation, becoming more active, provokes a flow of blood proportioned to the intensity of cerebral activity. In this respect, natural sleep and the anæsthetic sleep of choroform are alike; in both cases, the brain, sunk into rest or inactivity, presents the same paleness and relative bloodlessness.

The experiment is made in this manner: A part of the bony covering of an animal's skull is carefully removed, and the brain laid bare, so as to study the circulation at the surface of this organ. Then chloroform is administered to produce insensibility. In the first exciting stage of the action of the chloroform, the brain is observed to grow congested and to lap over the edges; but as soon as the stage of anæsthetic sleep is reached the substance of the brain sinks in and grows paler, presenting a languid movement of capillary circulation, which lasts as long as the state of sleep or cerebral rest continues. For the study of the brain in natural sleep, a circular trepan is made on a dog's head, and the piece of bone removed is replaced by a watch-glass carefully adjusted to the exact opening, so as to prevent the irritating action of the air. The animals subjected to the operation survive it; and observations on their brain through this sort of window, while awake and when asleep, prove that when the dog is asleep the brain is always paler, and that a fresh afflux of blood is regularly noticed on his awaking, when the functions of the brain resume their activity. Facts analagous to those observed in animals have been studied directly in the human brain.—*Boston Journal of Chemistry.*

*Oxygen in Scarlet Fever.*—Dr. Bayles (*N. Y. Med. Journal,* September, 1873) gives the results of his experience in the use of oxygen in scarlet fever, as follows: "It will tranquilize in one stage and energize in the other. In a surprisingly short time, with the free use of gas, I have seen the pulse brought down from high levels to very nearly the normal standard of health. In the same subject, at another time, the pulse small, thready, feeble and slow, would quikly be sent up to its normal range and volume. I have never seen a case succumb to the blood poisoning wherein this element has been used."

*Cerebro Spinal Meningetis.*—In the month of December last we encountered two cases of this protean malady, the symptoms and results of which confirm us in the opinion long entertained, that there are two distinct varieties of this affection, to-wit *inflammatory and non-inflammatory.*

The former variety is not so frequent as the latter. It is usually ushered in by a prolonged and violent chill, accompanied by congestion toward the cerebro spinal centers, followed by more or less febrile reaction, intense pain in the head, neck and back, redness of the eyes, the cerebral irritation and congestion tending rapidly to inflammation, effusion of lymph, delirum, coma and death. The poison in this variety seems first to impress the brain rather than the spinal column, and is sometimes so overwhelming that the patient sinks under the initial or congestive stage, before the inflammatory point is reached. Here we may have petecchial eruption and symptoms characteristic of *Spotted Fever.*

In other cases wherein more or less reaction occur, we sometimes have a specific eruption resembling measels, and occasionally mingled with urticaria. Three cases presenting this peculiarity occured in the vicinity of Atlanta during the past winter, all of which proved fatal. One of these cases came under our treatment. The reaction and fever was fully established. The eruption very much like measles, came out on the fourth day, accompanied with delirium, intense headache, and all the marks of cerebral inflammation with coma and death on the fifth day. Cold to the scalp, veratrum and bromide of potassium, and revulsives to the spine were the remedies used. In this we yielded to the now prevalent idea that the disease is non-inflammatory. We are now satisfied that the lancet in this case, ought to have been freely used early in the attack as inflammation or active congestion, which must lead to inflammation, undoubtedly exists in this variety of the affection.

In the non-inflammatory variety the poison imbibed appears to be less in quantity, or so modified that its effects are less active, resulting in a degree of *irritation* to the cerebro-spinal centers which, though sufficiently strong in many instances to cause rigidity of the post-cervical muscles, opistholonous, etc., yet fall short of developing inflammatory action, being analagous in its operations to strychnine poisoning, or tetanic spasms.

Of course the treatment in this class of cases must be different so far as depletory measures are concerned.

One of the cases to which we referred in the outset was of this type. The fever was comparatively light and remittent. There was headache and delirium, particularly at night, pain and rigidity of the neck, etc. It recovered in a few days.

Treatment—Sinapisms to spine, heat to the extremities, cups and cold water to the head, quinine in the morning, and the persevering use of the following preparation: ℞. Bromide potassium, tinc. gelseminum, tinc. deodorized opium, aa. ʒi. water; six table-spoonfuls. M. S. Take one table-spoonful every two or four hours.

*Oxygen in Opium Poisoning.*—Dr. Farrington (*Medical Times*, August 25, 1873) describes a case in which after the ènergetic use of coffee, atropia, artificial respiration, whisky and the galvanic battery, had each and all failed to restore consciousness, the administration of oxygen gas was followed by a gradual return to consciousness. Though the patient was attacked on the following day by double pneumonia, and ultimately died, the doctor says he is fully convinced of the value of this gas in cases of this sort. [We have seen the administration of oxygen to persons greatly intoxicated followed by the relief of all evidences subjective or objective of such intoxication. Several times we have been gratified with the ease by which oxygen removed the narcosis of chloroform or ether. This has been especially true of cases in which the anæsthetic acted badly, and unpleasant heart or lung symptoms appeared.—Eds.]—*Detroit Medical Review.*

*Remedy for Dyspepsia.*—℞. Manganesii iodidi, gr. xv.; ext. valerianæ, gr. xxx.; podophylline, gr. xv.; ext. barosmæ, gr. xxx.; ext. nuc. vomicæ, gr. xv.—M., et fiat in pilulas, xxx. S.—Take one a day; or, if the bowels are constipated, one night and morning.

Under the effects of this medicine, the stools will become natural, the tongue will clean, the urine will become clear, the acidity of the stomach will pass off, the skin will assume its hue of health, and the brain, heart, and nervous system will be relieved, provided the blood is properly oxidized, whilst digestion may be speedily restored by the use of the lacto-phosphate of lime, in most cases. Still, there will be found cases in which it will fail to produce the desired results, from the fact that it is not invariably sufficient without the aid of that particular organic principle denominated pepsin, which, with the presence of free lactic acid, is required in order to promote healthy digestion, especially of nitrogenized food.—*Physician's Monitor.*

*Epidèmic Neuralgia.*—From what we have observed in our practice during the winter, now closing, as well as from information derived from medical brethren in this vicinity, (DeCalb county, Georgia,) the prevalence of neuralgic affections has been unprecedented. Indeed the cases of neuralgia are, and have been so numerous that we are almost ready to proclaim it an epidemic,

*Barberry as an Antiperiodic.*—Piorry, Professor of Pathology at Paris, has demonstrated by a series of experiments that quinine and other febrifuges act by reducing the spleen and other enlarged visceria to the normal dimensions. Quinine had been recognized as possessing this inducing agency by Balley, Piorry's master, fifty years ago, but it was reserved for his pupil, to show that the same virtue resides in the *berberies vulgaris* or common *barberry.* He affirms that the superiority of this agent over quinine, as a febrifuge has been abundantly demonstrated in practice.

*Chloral Hydrate in Pertussis.*—Dr. Brynberg Porter, physician to the New York Free Dispensary for sick children, thus sums up his experience of chloral hydrate in pertussis, as exhibited at the dispensary:—"I am fully convinced of the marked effect of chloral hydrate in alleviating the symptoms of pertussis, and that there seems to be some evidence (though my number of cases is certainly very limited) to show that it has a positive effect in cutting short the disorder. It is the only remedy I have employed in this affection at the children's dispensary for some time."—*New York Medical Journal.*

*Tannin as an Antidote to Strychnine.*—Dr. Kursak, in the *Zeitschrift der Gesellschraft der Aerzte zu Wien,* has published a paper in which he claims tannin to be the most excellent antidote in strychnine poisoning, The tannate of strychnia formed, is insoluble in the intestinal juices. For one part of strychnia, twenty to twenty-five parts of tannin should be given, or even more, because a considerable part of the tannin is precipitated by the contents of the stomach—gelatine, for example. When tannic acid cannot be obtained, strong infusions of powdered gall-nuts, or green tea, should be used.—*Medical aud Surgical Reporter.*

---

